
JOURNAL INFORMATION
==============================
NLM Title Abbreviation: Wirtschaftsdienst
Journal ID: Wirtschaftsdienst
NLM Journal ID: 101086612

Wirtschaftsdienst (Hamburg, Germany : 1949)

ISSN: 0043-6275

ARTICLE INFORMATION
==============================
PMCID: PMC7560779
PMID: 33082602
DOI: 10.1007/s10273-020-2733-0
Article ID: 2733
Article version: 1
Subjects: Zeitgespräch

Perspektivische Auswirkungen der Corona-Pandemie auf die Wirtschaft und die Art des Arbeitens

Wintermann Ole ole.wintermann@bertelsmann-stiftung.de

Program Business in Society, Bertelsmann Stiftung, Carl-Bertelsmann-Straße 256, 33311 Gütersloh, Deutschland
Electronic publication date: 2020 Sep 25
Print publication date: 2020
Volume: 100
Issue: 9
First page: 657
Last page: 661
Copyright: © Der/die Autor(en) 2020
License: Dieser Artikel wird unter der Creative Commons Namensnennung 4.0 International Lizenz (https://creativecommons.org/licenses/by/4.0/deed.de) veröffentlicht.
Open Access wird durch die ZBW — Leibniz-Informationszentrum Wirtschaft gefördert.
License URL: https://creativecommons.org/licenses/by/4.0/

issue-copyright-statement: © The Author(s) 2020

==============================

Deutsche Unternehmen haben in den letzten Jahren die Digitalisierung von Arbeitsprozessen nicht entscheidend vorangebracht (Baethge und Boberach, 2018). Es waren und sind weiterhin typische Fehlermuster zu erkennen, wenn es darum geht, das eigene Unternehmen zu „digitalisieren“. Führungskräfte begriffen die Digitalisierung allzuoft allein als technische Herausforderung, was dazu führte, dass Zuständigkeiten allein in der IT-Abteilung verortet wurden. Dabei beschreibt die digitale betriebliche Transformation die qualitative Weiterentwicklung der vorhandenen Arbeitskultur in Kombination mit der beständigen Neuausrichtung des etablierten Geschäftsmodells. Beides wird durch eine entsprechende technisch fokussierte Digitalisierung natürlich garantiert. Ein technisch erfolgreiches Social Intranet ist aber erst dann funktional erfolgreich, wenn es durch eine Kultur der Offenheit und des Vertrauens mit Leben gefüllt wird.

Als Folge dieser technischen Fixierung und damit Fehleinschätzung haben Geschäftsführungen und Vorstände tendenziell gar nicht erkannt, welche Auswirkungen die Digitalisierung auf ihr Geschäftsmodell haben könnte. Die derzeitige Krise der deutschen Autoindustrie ist zu einem gewissen Teil dieser Unwissenheit geschuldet. Eine weitere Branche, die den Kunden und sein verändertes Kaufverhalten ignoriert hat, ist der Einzelhandel. Amazon ist nicht deshalb erfolgreich, weil Kunden in Deutschland den Fußgängerzonen bewusst den Rücken zukehren, sondern weil deutsche Einzelhandelsunternehmen es bis heute versäumt haben, die immense Bedeutung der Plattformökonomie zu erkennen und sich nach den veränderten Kundenwünschen auszurichten. Die Erstellung einer Verkaufsplattform im Internet und die Disruption angestammter analoger Geschäftsmodelle ist weniger eine technische als vielmehr eine kulturelle Herausforderung.

## Personalabteilungen arbeiten systemerhaltend

Die Personalabteilungen haben sich oftmals als unfähig erwiesen, dieser Kulturveränderung mit einer veränderten Personalpolitik Rechnung zu tragen. Die notwendige Änderung der Arbeitskultur – häufig subsumiert unter dem Oberbegriff New Work – wurde damit durch eine neue innere Haltung der neuen Beschäftigten in den Unternehmen nicht gestützt. Die Pfadabhängigkeit der Personalabteilungen, die ihnen durch die Geschäftsführungen und der internen Führungskultur vorgegeben wurde, führt zum Selbsterhalt eines in digitaler Perspektive nicht mehr funktionalen Systems.

## Digitalkompetenz in Führungsetagen nicht selbstverständlich

Transformation ist eine Führungsaufgabe. Wenn die Führung eines Unternehmens kein Signal zur Transformation gibt und intern ein Klima der Versagensangst, des Misstrauens, der Machtanhäufung durch intransparente Information und Kommunikation herrscht, wird die Belegschaft auch nicht bereit sein, diesen Schritt zu gehen. Warum sollten Informationen geteilt werden, wenn das Vorenthalten von Information durch Machtzuwachs gefördert wird? Das bedeutet aber im Umkehrschluss, dass der Erfolg der Transformation meist von der digitalen Kompetenz der Leitungsebene abhängig ist. Verschiedene Studien und Umfragen haben gezeigt, dass Führungskräfte sich hierbei selbst über- und die Angestellten unterschätzen (Grzymek und Wintermann, 2020).

Wenn diese Kompetenz nicht vorhanden ist, kann die digitale Transformation an Kleinigkeiten scheitern. Wenn in der Kommunikationsabteilung eines großen Unternehmens bisher eine Kultur des Sendens gegenüber den Kunden dominiert hat, wird die Fähigkeit zum Dialog mit dem Kunden auf Augenhöhe nicht von heute auf morgen erreicht werden. Wer soll intern aber dieses Defizit erkennen und benennen, wenn digital-soziale Kommunikation im Unternehmen auf den führenden Ebenen gar nicht verstanden wird?

### Die digitale Arbeitskultur wird nicht vorgelebt

Die fehlende Digitalkompetenz (als Selbstverständlichkeit) führt zudem dazu, dass Merkmale von New Work nicht erfolgreich skalieren können. Da Personalabteilungen durch ihre pfadabhängige Personalpolitik keine neuen Impulse für eine veränderte Arbeitskultur von außen einbringen können, ist der Wandel von internen Rollenmodellen abhängig, die es wagen, aus tradierten Prozessen und Rollen auszubrechen. Wesentliche Elemente von New Work (es gibt einige unterschiedliche Definitionen) sind die Kommunikation auf Augenhöhe, die Fähigkeit zur sozialen Kollaboration, die Selbstvergewisserung der eigenen Haltung zu bestimmten Werten, die Kenntnis dessen, was einen selbst beruflich antreibt, die Offenheit für beständige Änderungen des Arbeitsumfeldes, die Bereitschaft, neue Wege zu gehen. Im denkbar schlechtesten Fall veröffentlicht die Geschäftsführung eine Einladung, das neue Social Intranet für den „Austausch" zu nutzen und vergisst dabei, selbst mit den Menschen darüber zu kommunizieren oder missachtet eine möglicherweise vorhandene Misstrauenskultur im Unternehmen, die eine digital-basierte Kommunikation und Zusammenarbeit von vornherein ausschließt.

### Mobiles Arbeiten steht unter Generalverdacht

Diese Misstrauenskultur ist es dann auch, die es Millionen von Menschen bisher verwehrt hat, mobil oder auch von zuhause aus statt im monotonen Büro zu arbeiten (Initiative D21, 2020). Die Führungsebene ist sehr häufig von der Angst vor dem Kontrollverlust geplagt. Statt einer moderneren Zielorientierung bei Erbringung der Arbeitsleistung wird allzuhäufig noch auf den Stundenzettel geachtet. Gepaart ist dies meist mit dem Glauben daran, dass das Team ohne Führung einfach keine entsprechende Leistung erbringen kann. So führt diese Angst zur oberflächlichen Selbstvergewisserung der Unabkömmlichkeit in der eigenen Führungsrolle.

Dabei zeigt sich inzwischen immer stärker, dass es gerade die Führungsebene sein kann, die die Kommunikation und Leistungsfähigkeit des unterstellten Teams behindert. Verschiedene Studien (State of California, 1990) zeigen zudem bereits seit Ende der 1980er Jahre, dass mobiles Arbeiten betriebswirtschaftlich, volkswirtschaftlich, umweltpolitisch und bezüglich der Vereinbarkeit von Privat- und Berufsleben ganz neue Horizonte eröffnen könnte. Auch in der Corona-Zeit erstellte Studien zum reduzierten Stresslevel und zur erhöhten Produktivität im Homeoffice werden die Frage aufwerfen, ob nicht zukünftig vielmehr das Arbeiten im Büro einem Rechtfertigungszwang unterliegen müsste (Deutsche Angestellten-Krankenkasse, 2020).

Es hat sich also gezeigt, dass Unternehmen in Deutschland in den letzten Jahren mehrheitlich ihre digitalen Hausaufgaben nicht gemacht haben. In dem Zusammenhang sei darauf hingewiesen, dass „Industrie 4.0" ein Sonderfall der betrieblichen digitalen Transformation darstellt, der sich allein darauf bezieht, die Anwendung digitaler Werkzeuge in der Industrie zu skalieren. Hierbei belegt Deutschland im internationalen Vergleich durchaus immer wieder Spitzenplätze (Huawei, Handelsblatt Research Institute, 2016). Nur: Der für New Work absolut essenzielle Kulturaspekt spielt in dieser Art der Digitalisierung eine sehr nachrangige Rolle (Boes, 2019).

### Und dann kam Corona

Der Corona-Lockdown hat nun Anfang März im Deutschland in etlichen Unternehmen zu der Situation geführt, dass einzelne Elemente von New Work von einem Tag auf den anderen akzeptiert und eingeführt werden mussten. Gleichzeitig entstand für die darauffolgenden Wochen für einzelne Branchen wie den Einzelhandel, die Tourismus-Industrie, die Gastronomie und weitere Teile der Dienstleistungsbranche eine Krisensituation, da die Leistungen entweder nicht mehr nachgefragt wurden oder werden konnten. Es kann durchaus die These aufgestellt werden, dass ein großer Teil der Kurzarbeit oder der Entlassungen in Unternehmen der vorherigen Unfähigkeit zur digitalen Transformation des Geschäftsmodells geschuldet ist. Kurz gesagt: Führungsversagen hat die Arbeitslosigkeit und Kurzarbeit anscheinend unnötig stark ansteigen lassen (Boes, 2020). Oder anders formuliert: Unternehmen, die bereits vor Corona digital besser aufgestellt waren, werden die Krise, deren Ende ja noch nicht absehbar ist, wahrscheinlich besser überstehen können (Fraunhofer, DGPF, 2020)

### Corona und die Auswirkungen auf Branchen und Arten des Wirtschaftens

Nach unserer aktuellen Expertenbefragung (Bertelsmann Stiftung, Münchner Kreis, 2020) ist zu erwarten, dass die Luftfahrtindustrie, die Gastronomie, die Automobilindustrie, der Tourismus und der Kulturbereich durchaus auch langfristig zu den Verliererbranchen zählen werden, während der Bildungsbereich, die Telekommunikation, das Gesundheitswesen und die Pharma-Branche auf der Gewinnerseite stehen werden. Die Auswirkungen auf den Transportbereich sind unklar.

Wichtig zu unterscheiden gilt es bei dieser Analyse nach technischen und kulturellen Gründen dafür, auf welcher Seite eine Branche langfristig stehen wird. Zu den technischen Gründen zählt beispielsweise die Unmöglichkeit, Flugreisen anzutreten oder der Verlust von Kaufkraft durch Kurzarbeit. Die Gastronomie hätte die Möglichkeit gehabt, zumindest einen Teil der Verluste durch Anbindung an Lieferplattformen auszugleichen (was einzelnen gastronomischen Betrieben auch gelungen ist). Der Einzelhandel hätte, wenn es denn eine deutsche gemeinsame Lieferplattform gegeben hätte, einen beträchtlichen Teil der Umsätze, die nun zu Amazon abgewandert sind, bei sich halten können. Große Unternehmen, die ihren Mitarbeitenden bisher mobiles Arbeiten verweigert hatten, konnten diesen nun nicht im ausreichenden Maße die Soft- und Hardware-Infrastruktur zur Verfügung stellen.

Langfristig relevant werden aber vor allem kulturell induzierte Veränderungen sein. So erlebt die Autoindustrie trotz Wiederanfahren der Verkäufe bei den Händlern eine nachhaltigen Nachfrageflaute, die wohl nicht allein der Krisensituation geschuldet ist. Gleichzeitig erlebt die Fahrrad-Branche einen nie erlebten Boom. Auf der Werteebene könnten sich hier eventuell langfristig wirkende Veränderungen des Wertesystems der Kunden andeuten. So glaubt jeder dritte befragte Experte, dass das Reisen insgesamt und auf Dauer reduziert wird. Home Schooling, gegenseitige Wertschätzung, Entschleunigung, die Anwendung von Künstlicher Intelligenz für den Gesundheitsbereich und mögliche Katastrophenszenarien sowie die Digitalisierung des Bildungssystems sind Trends, die als übergeordnet und beständig identifiziert werden. Dass Corona sogar die Wirtschaftsordnung auf Dauer verändern wird, glaubt immerhin jeder vierte befragte Experte (Krcmar und Wintermann, 2020).

## Corona und die Auswirkungen auf die Art des Arbeitens

Auf der individuellen Arbeitsplatzebene deuten sich ebenfalls langfristig wirksame Veränderungen an. Die befragten Experten haben angegeben, dass sie bereits gegenwärtig in der Krisenzeit Veränderungen der internen Kommunikation durch Verwendung digitaler Werkzeuge, der Zusammenarbeit und des Verhaltens der Mitarbeite sowie der Kundenkommunikation wahrnehmen. Stabs- und Backendbereiche wie Controlling, Management und Führung sowie Vertrieb werden als weniger stark betroffen angesehen. Befragt nach den langfristig wirksamen Veränderungen verweisen die Experten auf die allgemeine Digitalisierung von Dienstleistungen und Kundenkommunikation. Zwei von drei Experten gehen davon aus, dass sich die Präsenzkultur in deutschen Büros auflösen wird. 84 % der Experten meinen, dass Homeoffice ein bestimmender Trend in der Arbeitswelt sein wird. Von einer deutlichen Mehrheit der Befragten wird geäußert, dass Video-Conferencing weite Teile der Präsenzformate ablösen wird, dass das Arbeitsleben räumlich und auch zeitlich flexibler werden wird und dass dies ein neues Austarieren von Privat- und Berufsleben mit sich bringen muss. Ebenfalls eine Mehrheit meint, dass sich das Führungsverhalten weg von Kontrolle und hin zu Vertrauen entwickeln wird.

Dass demnach die Corona-Krise die digitale betriebliche Transformation beschleunigen wird, glauben 92 % der befragten Experten. Betrachtet man diese Erwartungshaltung gemeinsam mit den Zahlen zur betrieblichen digitalen Baustelle in Unternehmen hierzulande, wird der immense Handlungsdruck deutlich. Die Krise zu bekämpfen, heißt demnach auch, die digitale Transformation gerade jetzt voranzutreiben. Die beobachtbaren tradierten und vom Controlling getriebenen Verhaltensmuster, die darauf abzielen, finanzielle Ressourcen dementgegen einzusparen, verheißen für Deutschland nichts Gutes. Dies macht sich auch bei volkswirtschaftlichen Szenarien zur weiteren konjunkturellen Entwicklung bemerkbar.

## Corona und die Volkswirtschaften: L, V oder W?

Um die Komplexität der Pandemiefolgen für Manager und Entscheider zu reduzieren, ist es populär geworden, die denkbaren volkswirtschaftlichen Szenarien für die nächsten Monate in Form von Buchstaben zu kommunizieren. Hierbei steht das „V“-Szenario für eine schnelle konjunkturelle Erholung (Aufschwung). Zugrunde liegt diesem Szenario die Vorstellung einer baldigen Rückkehr zur Normalität, die es Unternehmen ermöglicht, wieder in gewohnte Produktionsprozesse und globale Lieferketten einzusteigen. Im besten Fall wurde die Krise genutzt, um tradierte Prozesse und Wege zu überdenken oder sogar an veränderte globale Produktionsbedingungen anzupassen. Positiv verstärkend kommt eine Revitalisierung des Gemeinschaftsgefühls durch die Erfahrungen der gegenseitigen Hilfe dazu.

Im „L“-Szenario bedroht eine zunehmende Zahl von Insolvenzen den sozialen und wirtschaftlichen Kitt der Gesellschaft. Es besteht die Gefahr nachhaltig wirkender gesamtgesellschaftlicher Krisen auf verschiedenen Systemebenen, die sogar die Demokratie gefährden können. Damit einher geht das Risiko eines Rückzugs auf die Ich- und nationale Ebene des Individuums, des Staates. Die systemrelevante Negativität verhindert eine wirtschaftliche Erholung.

Im „W“-Szenario folgt auf eine erste schnelle Erholung eine zweite Krisenwelle. Während in der ersten Welle noch ausreichend Ressourcen zur Verfügung standen, um die Pandemiefolgen finanziell und sozial abzufedern, fehlen diese Ressourcen in der zweiten Pandemiewelle. Folge sind zunehmende Marktbereinigungen (aka Insolvenzen) und der Versuch des Staates, ein Mindestmaß an politischer, wirtschaftlicher und sozialer Sicherheit zu garantieren.

Während das V-Szenario – gerade auch in der Zusammenschau mit Konjunkturpaketen, die die Nachhaltigkeit des Wirtschaftens voranbringen könnten – überwiegend ein positives chancenorientiertes Potenzialszenario abbildet, steht das L-Szenario genau für das Gegenteil, während das komplexe W-Szenario mit unsicherem Ausgang wahrscheinlich die Realität am ehesten treffen dürfte.

Diese Szenarien finden sich inzwischen in etlichen Publikationen von Beratungsunternehmen, die eben auch auf die entsprechende Zielgruppe zugeschnitten sind. So verwundert es nicht, dass eine rein volkswirtschaftliche Perspektive die Debatte um die Corona-Krisenzeit dominiert. Es ist methodisch gesehen der große Fehler, die Zukunft der Post-Corona-Gesellschaft ganz überwiegend an der Entwicklung des Bruttoinlandsprodukts festzumachen. Damit begeben wir uns in eine Abhängigkeit, deren fatale Auswirkung auf das Erdklima und den vermeintlichen „Wohlstand" der Menschen ja erst durch Corona so deutlich geworden ist (Universität Sidney, 2020). Die methodischen und fachlichen Scheuklappen verhindern also trotz der Krisenzeit ein Ausbrechen aus gewohnten Denkmustern und verstetigen entgegen den Notwendigkeiten weiter die Pfadabhängigkeiten.

## Betriebliche digitale Transformation kann kein Selbstzweck sein

Das gerade in Deutschland dringend benötigte Nachholen der betrieblichen digitalen Transformation sollte (Hägelen et al., im Erscheinen) aus den genannten Gründen ganzheitlich und interdisziplinär angegangen werden. Corona bietet die Chance, das Geschäftsmodell, die tradierten Logiken und Entscheidungswege, den Antrieb des Unternehmens, wie den inneren Antrieb der Menschen selbst, die Auswirkung der Geschäftstätigkeit auf die Umwelt und das Verständnis, dass auch Unternehmen Teil eines ganzheitlichen Systems sind, das sich eben nicht der Wirkung einer Pandemie in einem Vakuum entziehen kann, auf den Prüfstand zu stellen.

Zuerst sollten betriebliche Entscheider fragen, wo – im inhaltlichen, prozeduralen und örtlichen Sinne – die digitale Transformation eigentlich beginnt. Ist die unternehmensweite Umstellung auf Homeoffice nicht schon ein wichtiger Teil der Transformation? Ist statt Homeoffice nicht vielmehr die Rückkehr zur standardisierten Office-Arbeit der Ausnahmefall? Um diese Frage stellen zu können, bedarf es einer weitsichtigen Geschäftsführung, die nicht davor zurückscheut, auch eigene Privilegien und „Wahrheiten" kritisch zu hinterfragen.

Es kommt darauf an, dass diese Geschäftsführung sodann „Keimzellen" der Transformationsbereitschaft in der Belegschaft identifiziert und mit Ressourcen und einem freien Rücken ausstattet, damit diese Freiheit zu innovativen Denkansätzen führt. In der Folge werden Rollen, Führungsebenen, Organisationsformen angepasst oder gestrichen werden; eine Digitalisierung im „Frontend", ohne dass dies Auswirkungen auf die Situation im „Backend" hätte, kann es nicht geben. Begleitet werden muss dieses digitale Experiment mit einer veränderten Arbeits- und Führungskultur. Auch hier zeigt die Corona-Krisenzeit, welche Führungskraft kein Problem mit der digitalisierten Rolle von „Führung" hat. Es gilt, diese Personen auch in der Zeit nach der Krise weiter zu fördern. Die vielzitierte Fehlerkultur, die Kommunikation auf Augenhöhe, die Agilität des Projektmanagements, der Umgang der lebenden Organisation mit der komplexen Umwelt sind nur bei einem veränderten Führungsverhalten erreichbar.

Die Ganzheitlichkeit des Ansatzes zur erfolgreichen betrieblichen Transformation wird sichtbar, wenn man sich die betroffenen Transformationsbereiche nochmals vor Augen führt: Es geht um die reine Technisierung der Arbeit und gleichzeitig auch die Anpassung des Geschäftsmodells an digitale Plattformlogiken, die Neuaufstellung der Arbeitsorganisation in Hinblick auf digitale Kommunikation und Kollaboration, die Implementierung einer komplett neuen digitalen Arbeitskultur durch Vorleben der Führung und um den Kompetenzaufbau und die Bereitschaft zum beständigen Lernen von Digitalkompetenzen sowohl der Führenden als auch der Geführten. Vielleicht sollten wir bei der Gelegenheit auch die tiefergehende implizite Wertung des Begriffs „Führung" überdenke, geht es doch nicht um Schafe, die eine Führung benötigen, sondern vielmehr um die Zusammenarbeit von mündigen Arbeitnehmern.

## Fazit

Die Corona-Krise dynamisiert eine seit Jahren bestehende Notwendigkeit der flächendeckenden digitalen betrieblichen Transformation. Die Hauptgefahr besteht nicht etwa nur in der ausbleibenden Nachfrage nach Gütern und Dienstleistungen, sondern vor allem auch darin, dass Geschäftsführungen nicht erkennen, dass diese Krisenzeit eine große Chance zum Aufbruch darstellt. Hierbei muss man nicht unbedingt gleich soweit gehen, grundsätzlich einzufordern, unsere umweltzerstörerische Wirtschaftsweise zu überdenken; sehr viel eher geht es momentan um die Chance zur krisenbedingten Neuausrichtung, um das W- und das L-Szenario unwahrscheinlicher werden zu lassen. Die Blaupause für eine erfolgreiche Transformation liegt – wissenschaftlich überprüft – vor. Nun ist es an den Entscheidern in der Wirtschaft, diese Blaupause für sich und ihr Unternehmen nutzbar zu machen.

Dr. Ole Wintermann, Senior Project Manager, Program Business in Society der Bertelsmann Stiftung in Gütersloh.


Literatur

Baethge, C. B. und M. Boberach (2018), Zukunft der Arbeit in deutschen KMU- Werkstattbericht, Kantar TNS, Bertelsmann Stiftung.
Bertelsmann Stiftung und Münchner Kreis e. V. (2020), Studie zu den Auswirkungen der Corona-Pandemie in gesellschaftlicher, wirtschaftlicher und technologischer Hinsicht, im Rahmen der Münchner Kreis Zukunftsstudie Band VIII: Leben, Arbeit, Bildung 2035+.
Boes, A. (2020): It’s the internet, stupid! Deutsche Wirtschaft im Paradigmenwechsel im IdGuZdA Blog, 4. Dezember 2019, https://idguzda.de/blog/openspace4future (23. Juli 2020).
Deutsche Angestellten-Krankenkasse, (2020), Digitalisierung und Homeoffice entlasten Arbeitnehmer in der Corona-Krise, 22. Juli 2020, https://www.dak.de/dak/bundesthemen/sonderanalyse-2295276.html#/ (23. Juli 2020).
Fraunhofer IAO in Kooperation mit der Deutschen Gesellschaft für Personalführung DGPF (2020), Arbeiten in der Corona-Pandemie — Auf dem Weg zum New Normal, Düsseldorf.
Grzymek, V. und O. Wintermann (2020), Wie digital sind die Unternehmen in Deutschland? — Ergebnisse einer repräsentativen Befragung unter Erwerbstätigen, Kantar, Bertelsmann Stiftung.
Huawei und Handelsblatt Research Institute (2016), Industrie 4.0 im internationalen Vergleich — Vergleich der Industrie 4.0-Wettbewerbsfähigkeit Chinas, Deutschlands, Japans und der USA, Düsseldorf.
Initiative D21 (Hrsg., 2020), D21 Digital Index 19/20- Wie digital ist Deutschland?, Berlin.
Krcmar, H. und O. Wintermann (2020), Begleittext, in Bertelsmann Stiftung, Münchner Kreis, TUM Campus Heilbronn (Hrsg.), Studie zu den Auswirkungen der Corona-Pandemie in gesellschaftlicher, wirtschaftlicher und technologischer Hinsicht, im Rahmen der Münchner Kreis Zukunftsstudie Band VIII: Leben, Arbeit Bildung 2035+, https://www.bertelsmann-stiftung.de/fileadmin/files/user_upload/Sonderstudie_Corona_Begleittext_final.pdf (9. September 2020).
The State of California (1990), The State of California Telecommuting Pilot Project, Final Report, Juni 1990.
Universität Sidney (2020), Socio-economic, environmental impacts of COVID-19 quantified, 10. Juli 2020, https://www.sydney.edu.au/news-opinion/news/2020/07/10/socioeconomic-and-environmental-impacts-of-coronavirus-quantifie.html (23. Juli 2020).
